# Acupuncture for Poststroke Shoulder Pain: A Systematic Review and Meta-Analysis

**DOI:** 10.1155/2016/3549878

**Published:** 2016-07-31

**Authors:** Sook-Hyun Lee, Sung Min Lim

**Affiliations:** Department of Clinical Research on Rehabilitation, Korea National Rehabilitation Research Institute, Seoul, Republic of Korea

## Abstract

*Objective*. To summarize and evaluate evidence for the effectiveness of acupuncture in relieving poststroke shoulder pain.* Methods*. Seven databases were searched without language restrictions. All randomized controlled trials that evaluated the effects of acupuncture for poststroke shoulder pain compared with controls were included. Assessments were performed primarily with the Visual Analogue Scale (VAS), Fugl-Meyer Assessment (FMA), and effective rates.* Results*. In all, 188 potentially relevant articles were identified; 12 were randomized controlled trials that met our inclusion criteria. Meta-analysis showed that acupuncture combined with rehabilitation treatment appeared to be more effective than rehabilitation treatment alone for poststroke shoulder pain, as assessed by VAS (weighted mean difference, 1.87; 95% confidence interval [CI], 1.20–2.54; <0.001); FMA (weighted mean difference, 8.70; 95% CI, 6.58–10.82; *P* < 0.001); and effective rate (RR, 1.31; 95% CI, 1.18–1.47; *P* < 0.001).* Conclusions*. Although there is some evidence for an effect of acupuncture on poststroke shoulder pain, the results are inconclusive. Further studies with more subjects and a rigorous study design are needed to confirm the role of acupuncture in the treatment of poststroke shoulder pain.

## 1. Introduction

Globally, stroke is the second most common cause of death and a major cause of disability [1]. Shoulder pain is a commonly seen disability during the subacute recovery phase in stroke patients [2]. The other types of shoulder injuries include glenohumeral subluxation, rotator cuff tears, brachial plexus injury, spasticity of shoulder muscles, soft-tissue trauma, and shoulder-hand syndrome [3]. Shoulder pain can interfere with the recovery of arm function, regular rehabilitation, and independence in activities of daily living [4]. Poststroke shoulder pain has an estimated prevalence of 22%-23% among the general population of stroke patients and is seen in 54%-55% of stroke patients in rehabilitation settings [5].

Despite several available treatment options such as nonsteroidal anti-inflammatory drugs (NSAIDs), corticosteroid and Botulinum toxin type A injections, nerve blocks, exercise, strapping, and electrical stimulation for patients with poststroke shoulder pain, it has been estimated that 30%–50% of such patients do not experience pain relief [6]. Therefore, there is a need for complementary and alternative treatments that are effective in relieving poststroke shoulder pain.

Acupuncture has been used since the ancient times to treat various clinical conditions, pains, musculoskeletal problems, and neurologic disorders [7]. Many published clinical studies, including randomized controlled trials (RCTs), have explored acupuncture as a treatment option for poststroke shoulder pain, and most reports have shown positive clinical effects of acupuncture in the treatment of poststroke shoulder pain. A recent systematic review has examined the efficacy of acupuncture in the treatment of poststroke shoulder pain [8]. However, to the best of our knowledge, a meta-analysis has never been conducted on this topic. In the current review, we have assessed the effectiveness of acupuncture in treating stroke patients with poststroke shoulder pain by using a meta-analysis.

## 2. Methods

### 2.1. Search Methods for Identification of Studies

The search was performed without language and publication year restriction. We searched Medline, EMbase, and the Cochrane Central Register of Controlled Trials from database inception till March 2015. For Korean publications, we searched three Korean medical databases—Research Information Service System, National Discovery for Science Leaders, and OASIS. For Chinese articles, we searched the China National Knowledge Infrastructure (CNKI) database. A hand search of relevant references from previous systematic reviews was conducted.

The keywords used for the search were “stroke OR apoplexy OR cerebral infarction OR cerebral hemorrhage” AND “acupuncture OR electroacupuncture” AND “shoulder pain” in each database language. The search strategy was adjusted for each database.

### 2.2. Inclusion/Exclusion Criteria

Relevant clinical trials were included if the following criteria were met: (1) the study was a randomized, controlled trial (RCT); (2) the study included patients diagnosed with poststroke shoulder pain; (3) patients with poststroke shoulder pain at baseline were enrolled; and (4) poststroke shoulder pain was an outcome measure of the study.

Trials were excluded if the study design did not allow evaluation of acupuncture effects on poststroke shoulder pain; that is, studies were excluded if they (1) compared different types of acupuncture, (2) adopted complex treatment without examining the effects of acupuncture alone, or (3) reported insufficient information.

### 2.3. Data Extraction

Two reviewers (L. S. H. and L. S. M.) independently reviewed the extracted data from each paper using a standardized data extraction form and reached consensus on all items. Extracted data included authors, year of publication, sample size, interventions, main outcomes, and adverse events. The main outcomes used in this systematic review were the Visual Analogue Scale (VAS), the Fugl-Meyer Assessment (FMA), and effective rates. The primary outcome referred to the intensity of shoulder pain evaluated through VAS [9]. VAS is most commonly anchored by “no pain” (score of 0) and “pain as bad as it could be” or “worst imaginable pain” (score of 10 [10 cm scale]). It has been shown to be a reliable measure, and there is a valid correlation between vertical and horizontal orientations [10, 11]. The secondary outcome assessments used were the Fugl-Meyer Assessment of upper extremity (FMA-UE) and effective rates. FMA-UE is a measure of motor recovery after stroke and has a range of 0–2, with a total score ranging from 0 to 66. It has been shown to be reliable and to have a valid correlation coefficient [12–14]. The effective rate was calculated based on the proportion of effectively treated patients (complete or partial improvement) to the proportion of patients in whom treatment was ineffective (no improvement).

### 2.4. Quality Assessment

The two reviewers independently assessed the methodological quality and the risk of bias of the included studies by using the risk of bias (ROB) tool in the Cochrane Handbook for Systematic Reviews of Interventions (Version 5.0.2). This instrument consists of 8 domains: random sequence generation; allocation concealment; blinding of patients, personnel, and outcome assessors; incomplete outcome data; selective outcome reporting; and other sources of bias. The tool ranks evidence from research studies as having “high,” “low,” or “unclear” levels of bias; it is also appropriate for evaluating the methodological quality of RCTs. In cases in which the reviewers' opinions differed, a joint opinion was reached through discussion.

### 2.5. Statistical Analysis

All statistical analyses were performed with the Reviewer Manager Software, version 5.3 (Cochrane Collaboration, Oxford, UK). Summary estimates of treatment effects were calculated using a random-effects model. The impact of acupuncture on dichotomous data was expressed as the risk ratio (RR); for continuous outcomes, the mean difference was calculated with a 95% confidence interval (CI). We assessed the clinical and methodological heterogeneities of the enrolled studies according to the subgroup analysis that was performed. The statistical heterogeneity in the subgroups was analyzed using the *I* ^2^ test and was considered significant when *I* ^2^ was greater than 50%. Even when a low heterogeneity was detected, a random-effects model was applied, because the validity of tests of heterogeneity can be limited with a small number of component studies. Publication bias was not a factor because of the limited number of studies.

## 3. Results

### 3.1. Study Description

**Figure 1:**
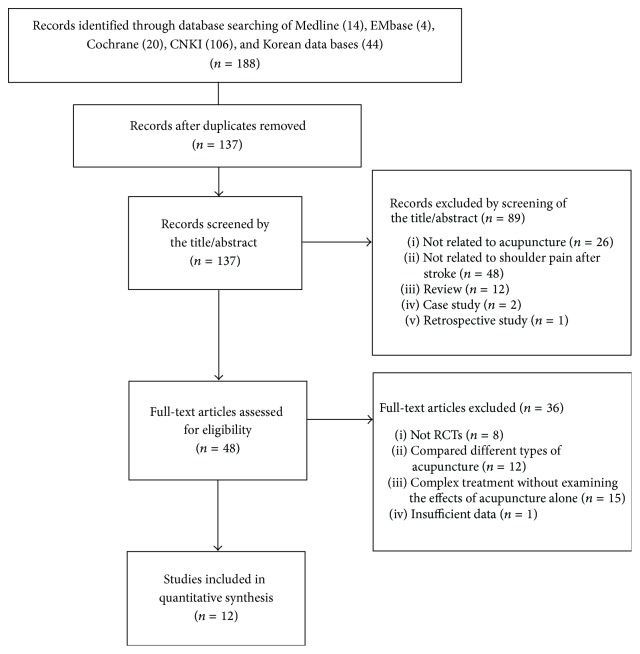
Flow chart of the trial selection process.

We identified 188 publications; 12 met the eligibility criteria (Figure 1). The articles included in the analysis are summarized in Table 1. The 12 articles were published from 2002 to 2014. All 12 studies were from China. The languages of the publications were English and Chinese.

### 3.2. Descriptions of Acupuncture Treatment

The majority of the included RCTs stated that the rationale for acupuncture point selection was drawn from Traditional Chinese Medicine theory (Table 1) [15–26]. Eleven studies used acupuncture treatment [15–19, 21–26], and one study used electroacupuncture treatment for poststroke shoulder pain [20]. In all, 32 acupuncture points were used for the treatment of poststroke shoulder pain. Quchi (LI-11) was most often used as acupoints for poststroke shoulder pain treatment [15, 17, 20, 23, 25, 26].

### 3.3. Meta-Analysis

**Figure 2:**
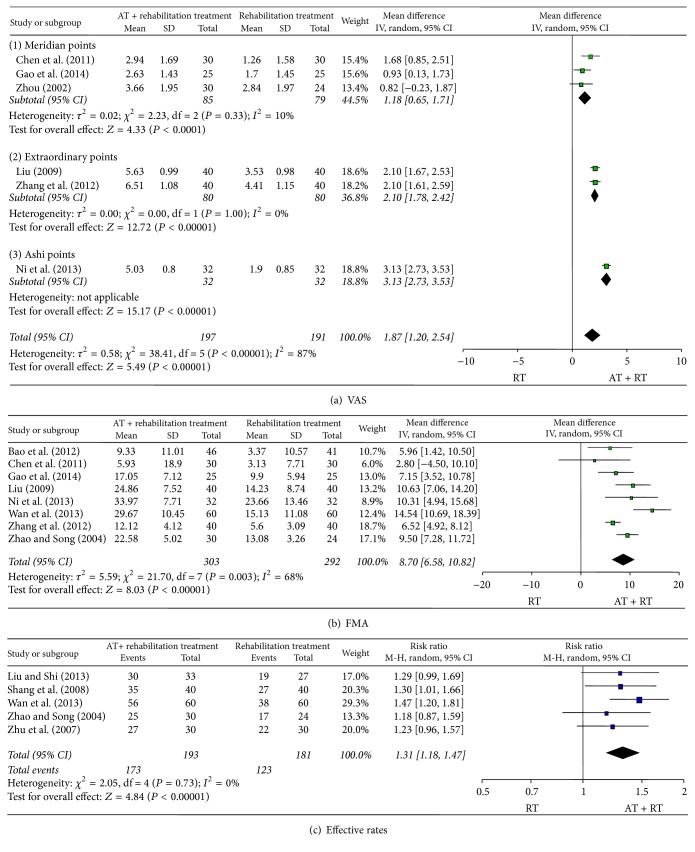
Meta-analysis of acupuncture for poststroke shoulder pain according to the different assessment tools.

We conducted a meta-analysis of the study results based on the pain assessment scales used (Figure 2). In 6 studies that used the VAS to assess treatment results, we found that acupuncture combined with rehabilitation seemed more effective than rehabilitation alone for treatment of poststroke shoulder pain (weighted mean difference, 1.87; 95% confidence interval [CI], 1.20–2.54; *P* < 0.001; *n* = 388; *I* ^2^ = 87%). The subgroup analysis based on the type of acupuncture points used revealed that acupuncture combined with rehabilitation significantly reduced poststroke shoulder pain using meridian points (weighted mean difference, 1.18; 95% CI, 0.65–1.71; *P* < 0.001; *n* = 164; *I* ^2^ = 10%), extraordinary points (weighted mean difference, 2.10; 95% CI, 1.78–2.42; *P* < 0.001; *n* = 160; *I* ^2^ = 0%), and Ashi points (weighted mean difference, 3.13; 95% CI, 2.73–3.53; *P* < 0.001; *n* = 64).

In 8 studies that used FMA to compare the effects of acupuncture combined with rehabilitation versus rehabilitation alone, it was seen that the combination treatment had a significant effect on poststroke shoulder pain (weighted mean difference, 8.70; 95% CI, 6.58–10.82; *P* < 0.001; *n* = 595; *I* ^2^ = 68%).

In 5 studies that used the effective rate for a similar comparison, acupuncture had a significant effect in reducing poststroke shoulder pain (RR, 1.31; 95% CI, 1.18–1.47; *P* < 0.001; *n* = 374; *I* ^2^ = 0%).

### 3.4. Study Quality

The ROB results are shown in Table 2. With regard to random sequence generation and allocation concealment, 7 studies had a low ROB [15–19, 21, 22] and 5 studies had an unclear ROB [20, 23–26]. With regard to blinding of patients, one study had a low ROB [16] and 11 studies had an unclear ROB [15, 17–26]. Twelve studies had a low ROB with respect to incomplete outcome data [15–26] and all studies had a low ROB with respect to selective outcome reporting [15–26]. All studies had an unclear ROB with respect to other biases [15–26].

## 4. Discussion

Our systematic review and meta-analysis suggested evidence for the effectiveness of acupuncture in treating poststroke shoulder pain. Among the 188 studies retrieved, only 12 met the inclusion criteria for this meta-analysis. All 12 studies were RCTs performed in China and all showed favorable results for combined acupuncture and rehabilitation treatment compared with rehabilitation alone. Pain relief and improvement of upper-limb motor function are important treatment aspects in stroke rehabilitation. For outcome measurement, 6 studies used the VAS to evaluate pain intensity on a scale of 0–10. In 8 studies, the FMA was used to assess the upper-limb motor function improvement [27, 28].

Shoulder pain is a common problem following a stroke, and 75% of patients complain of pain in the first 12 months after a stroke. This interferes with activity, recovery, and rehabilitation [3, 29]. It requires a coordinated multidisciplinary pain management approach to minimize interference with rehabilitation and optimize outcomes [30].

Acupuncture is an effective treatment for chronic pain in many patients [8]. An NIH consensus report also stated that the incidence of adverse effects of acupuncture is substantially lower than that of many other accepted medical interventions [31]. In the current study, we too did not report any adverse acupuncture-related events in patients with poststroke shoulder pain.

This review also had certain limitations. Most importantly, some of the included studies were of poor quality and had methodological shortcomings such as an inadequate level of blinding. Even though it is difficult to blind the acupuncture therapist to the patient, attempts should have been made to blind the patients and outcome assessors in order to minimize the performance bias. Most of the included studies had an unclear risk of bias for patient blinding, and a preponderance of positive results was demonstrated. Future studies should include a sham treatment group as a control to exclude a substantial placebo effect. Although we did not register or publish the study protocol earlier, it is recommended that the protocol be registered or published to avoid duplication and reduce the risk of a reporting bias.

Future RCTs assessing the effectiveness of acupuncture for poststroke shoulder pain relief must overcome selection, performance, and detection biases. Hence, a large-scale, multicenter trial is recommended. There is a necessity for long-term follow-up studies to determine the efficacy and safety of acupuncture for patients with poststroke shoulder pain and the persistence of its effects. Clinical trials involving acupuncture must use an optimal form of treatment, defined by examining standard texts and by surveying and consulting experts [32]. Further, clinical studies should be reported by using the basic guidelines for reporting clinical trials such as the CONSORT statement and Standards for Reporting Interventions in Controlled Trials of Acupuncture (STRICTA) [33, 34]. It is possible to attain more robust conclusions on the efficacy of treatment to relieve poststroke shoulder pain through rigorous designs, reasonable appraisals, and critical analyses.

## 5. Conclusions

Although there is some evidence for an effect of acupuncture on poststroke shoulder pain, the results are not conclusive. Further studies with a larger number of subjects and a rigorous study design are needed to confirm the role of acupuncture in the treatment of poststroke shoulder pain.

## Figures and Tables

**Table 1:** Summary of randomized controlled trials of acupuncture for poststroke shoulder pain.

Author (year)	Condition sample size	Intervention group	Control group	Main outcomes
Gao et al. [15] (2014)	Poststroke shoulder pain (25/25/25)	(A) AT + rehabilitation treatment (LI15, SI9, TE14, SI11, HT1, LI11, LI10, PC6, TE5, and LI4; 6 times weekly for 4 weeks, 30 min)	(B) AT (C) Rehabilitation treatment	(1) VAS (2) FMA

Liu and Shi [16] (2013)	Poststroke shoulder pain (33/27)	(A) AT + rehabilitation treatment (BL20, BL21, ST36, ST40, SP10, GV20, and EX-HN1; once every day for 4 weeks, 30 min)	(B) Rehabilitation treatment	(1) Effective rate

Wan et al. [17] (2013)	Poststroke shoulder-hand syndrome (60/60)	(A) AT + rehabilitation treatment (LU9, ST36, GB39, TE5, LI10, LI11, and LI15; once every day for 4 weeks, 30~40 min)	(B) Rehabilitation treatment	(1) FMA (2) Effective rate

Ni et al. [18] (2013)	Poststroke shoulder pain (32/32)	(A) AT + rehabilitation treatment (Ashi points; once every day for 60 days, 5 min)	(B) Rehabilitation treatment	(1) VAS (2) FMA

Zhang et al. [19] (2012)	Poststroke shoulder pain (40/40)	(A) AT + rehabilitation treatment (BP-LE6; once every day for 3 weeks, 10~20 min)	(B) Rehabilitation treatment	(1) VAS (2) FMA

Bao et al. [20] (2012)	Poststroke shoulder pain (46/41/42)	(A) EA + rehabilitation treatment (LI15, TE14, SI9, LI14, LI11, LI10, and TE5; once every day for 30 days, 30 min, 100 A, 2 Hz)	(B) EA (C) Rehabilitation treatment	(1) FMA

Chen et al. [21] (2011)	Poststroke shoulder pain (30/30)	(A) AT + rehabilitation treatment (CV12, KI17, ST26, Shangfengshidian, and Shangfeng shiwaidian; once every day for 2 weeks, 30 min)	(B) Rehabilitation treatment	(1) VAS (2) FMA

Liu [22] (2009)	Poststroke shoulder-hand syndrome (40/40)	(A) AT + rehabilitation treatment (BP-LE6; once every day for 40 days, 30 min)	(B) Rehabilitation treatment	(1) VAS (2) FMA

Shang et al. [23] (2008)	Poststroke shoulder-hand syndrome (40/40/40)	(A) AT + rehabilitation treatment (LI15, Jianquan, TE14, HT1, LI14, LI11, PC6, and LI14; 3 times weekly for 4 weeks, 40 min)	(B) AT (C) Rehabilitation treatment	(1) Effective rate

Zhu et al. [24] (2007)	Poststroke shoulder subluxation (30/30)	(A) AT + rehabilitation treatment (LI15, SI9, TE14, TE13; once every day for 4 weeks, 30 min)	(B) Rehabilitation treatment	(1) VAS (2) Effective rate

Zhao and Song [25] (2004)	Poststroke shoulder-hand syndrome (30/24)	(A) AT + rehabilitation treatment (SI11, LI15, LI11, TE7, TE4, Jingbi, and EXUE9; once every day for 10 days, 20 min)	(B) Rehabilitation treatment	(1) FMA

Zhou [26] (2002)	Poststroke shoulder pain (50/50)	(A) AT + rehabilitation treatment (LI15, TE14, SI9, LI11, LI10, TE5, and LI4; once every day for 4 weeks, 20 min)	(B) Rehabilitation treatment	(1) VAS

AT: acupuncture treatment, EA: electroacupuncture, VAS: the Visual Analogue Scale, and FMA: the Fugl-Meyer Assessment; adverse effects were not reported for any study.

**Table 2:** Quality assessment of included studies.

	Gao et al. (2014) [15]	Liu and Shi (2013) [16]	Wan et al. (2013) [17]	Ni et al. (2013) [18]	Zhang et al. (2012) [19]	Bao et al. (2012) [20]	Chen et al. (2011) [21]	Liu (2009) [22]	Shang et al. (2008) [23]	Zhu et al. (2007) [24]	Zhao and Song (2004) [25]	Zhou (2002) [26]
(1) Was the method of randomization adequate?	L	L	L	L	L	U	L	L	U	U	U	U

(2) Was the treatment allocation concealed?	L	L	L	L	L	U	L	L	U	U	U	U

(3) Was the patient blinded to the intervention?	U	L	U	U	U	U	U	U	U	U	U	U

(4) Were the personnel blinded to the intervention?	U	U	U	U	U	U	U	U	U	U	U	U

(5) Was the outcome assessor blinded to the intervention?	U	U	U	U	U	U	U	U	U	U	U	U

(6) Were incomplete outcome data adequately addressed?	L	L	L	L	L	L	L	L	L	L	L	L

(7) Are reports of the study free of suggestion of selective outcome reporting?	L	L	L	L	L	L	L	L	L	L	L	L

(8) Was the study apparently free of other problems that could put it at a high risk of bias?	U	U	U	U	U	U	U	U	U	U	U	U

Based on the risk of bias assessment tool from the Cochrane handbook for systematic reviews of interventions, low risk of bias: L, high risk of bias: H, and unclear risk of bias: U.
